# New Trends for Antimalarial Drugs: Synergism between Antineoplastics and Antimalarials on Breast Cancer Cells

**DOI:** 10.3390/biom10121623

**Published:** 2020-12-01

**Authors:** Diana Duarte, Nuno Vale

**Affiliations:** 1 OncoPharma Research Group, Center for Health Technology and Services Research (CINTESIS), Rua Plácido da Costa, 4200-450 Porto, Portugal; dianaduarte29@gmail.com; 2 Faculty of Pharmacy, University of Porto, Rua Jorge Viterbo Ferreira, 228, 4050-313 Porto, Portugal; 3 Faculty of Medicine, University of Porto, Al. Hernâni Monteiro, 4200-319 Porto, Portugal

**Keywords:** breast cancer, drug synergism, antineoplastic drugs, drug repurposing, antimalarial drugs, combination therapy

## Abstract

Chemotherapy plays a key role in breast cancer therapy, but drug resistance and unwanted side effects make the treatment less effective. We propose a new combination model that combines antineoplastic drugs and antimalarials for breast cancer therapy. Cytotoxic effects of two antineoplastic agents alone and in combination with several antimalarials on MCF-7 tumor cell line was evaluated. Different concentrations in a fixed ratio were added to the cultured cells and incubated for 48 h. Cell viability was evaluated using MTT and SRB assays. Synergism was evaluated using the Chou-Talalay method. The results indicate doxorubicin (DOX) and paclitaxel (PTX) alone at concentrations of their IC_50_ and higher are cell growth inhibitors. Mefloquine, artesunate, and chloroquine at concentrations of their IC_50_ demonstrate anti-cancer activity. In combination, almost all antimalarials demonstrate higher ability than DOX and PTX alone to decrease cell viability at concentrations of IC_50_ and lower than their IC_50_. The combination of chloroquine, artesunate and mefloquine with DOX and PTX was synergic (CI < 1). The combination of DOX and mefloquine after 48 h incubation demonstrated the highest cytotoxicity against MCF-7 cells, and the combination of DOX and artesunate was the most synergic. These results suggest antimalarials could act synergistically with DOX/PTX for breast cancer therapy.

## 1. Introduction

Breast cancer is the most commonly diagnosed type of cancer in women and represents the second leading cause of death by cancer in women worldwide [1]. The main subtypes of breast cancer are estrogen receptor positive/progesterone receptor positive (ER+/PR+), HER-2 positive (HER2+), and triple negative (ER−, PR−, HER2−) [2]. It is estimated that 75% of all patients have tumors expressing the estrogen and/or progesterone receptors [3]. Surgical removal and chemotherapy plays a key role in breast cancer therapy, being responsible for the long-term survival of patients [4]. However, chemotherapy often has its effectiveness decreased due to the ability of tumor cells to offer intrinsic or acquired resistance to these therapeutic agents, making the treatment less effective [5]. Other problems related to chemotherapy involve side reactions, making cancer treatment less pleasant, and narrowing the therapeutic window of antineoplastics [6]. It is, therefore, very important to develop new methodologies and protocols to ensure the long-term survival and good life quality of these patients by increasing the therapeutic efficacy of these treatments, while reducing the doses and toxicity. Doxorubicin (DOX) is an antineoplastic belonging to the anthracycline class that is widely used in breast cancer [7]. Its mechanism of action is based on the intercalation with DNA, which leads to the cleavage of DNA by the enzyme topoisomerase II (TOP2) and consequently the cell cycle stops in the G2/M phase, resulting in cell death [8]. In addition, studies suggest that the interconversion of DOX by cells to a semiquinone metabolite, leads to the formation of ROS that interfere with lipid peroxidation and cause damage to DNA and cell membranes [8]. Doxorubicin is usually given in combination with other chemotherapy medicines and used as adjuvant, neo-adjuvant, and to treat advanced-stage breast cancer. However, the use of this drug in therapy is conditioned by its cardiotoxicity as well as by the resistance of the tumor cells, leading to a decrease in its effectiveness [9]. Paclitaxel (PTX) is another antineoplastic used in the treatment of advanced stage breast cancers and is a natural diterpene alkaloid, isolated from the *Taxus brevifolia* tree [10]. In addition to its use in breast cancer, PTX is also employed in the therapy of other types of cancers, such as ovary, lung, etc. [11]. The main target of PTX is β-tubulin, a protein responsible for stabilizing the microtubule polymers. When binding to β-tubulin, PTX prevents its breakdown and cells are blocked in phases G0/G1 and G2/M, which leads to tumor cells death [12]. Several studies suggest that PTX can lead to increased expression of the FOXO1 gene, which may be involved in the inhibition of androgen receptors [13]. PTX also causes an increase in IL-10, an anti-inflammatory cytokine, as well as an increase in the production of ROS, by increasing the activity of NADPH oxidase [14]. The solvents used to solubilize this drug make PTX difficult to tolerate while being given and patients usually need to pre-medications to minimize reactions to the solvents. Furthermore, it causes together severe side effects such as low white blood cell count, susceptibility to infections, allergic reactions, hair loss, vomiting, and diarrhea, among others [15]. 

Several strategies, such as targeted therapies and controlled drug delivery, have been emerging. Targeted therapies are rational strategies based on the overexpression of specific molecular targets in tumor cells [16]. The controlled drug delivery allows the effective transport of several therapeutic agents to the sites of action, allowing drugs to be concentrated in the desired location and reducing the administered dose [17]. More recently, drug combination has gained interest in the treatment of many advanced malignancies and involves drug cocktails of two or more drugs. These combinations are chosen due to their non-overlapping resistance mechanisms [18] and the drug interaction (synergy/antagonism) phenotype [19]. Different drugs in combination act on different metabolic pathways that can contribute to carcinogenesis, turning the treatment more effective than monotherapy [20,21,22,23]. The effect of the combined drugs can be even greater if the resulting effect is higher than the addition of their effects alone, i.e., if the two drugs are synergic. This strategy aims to decrease the required dose to achieve a therapeutic effect and consequently reduce systemic toxicity [24]. It is therefore important to define how the drugs are combined, such as the molar ratio of each drug in the combination and precision is needed in translating these synergistic drug combinations into clinical practice [25]. The combination of two antineoplastic agents, such as DOX and cisplatin, has shown synergistic effect in phase III clinical trials; however this combination exhibited severe side-effects and drug resistance in clinical practice [26,27]. Combination regimens for breast cancer have also been evaluated in the clinic but toxicity has limited their application [28]. 

**Scheme 1:**
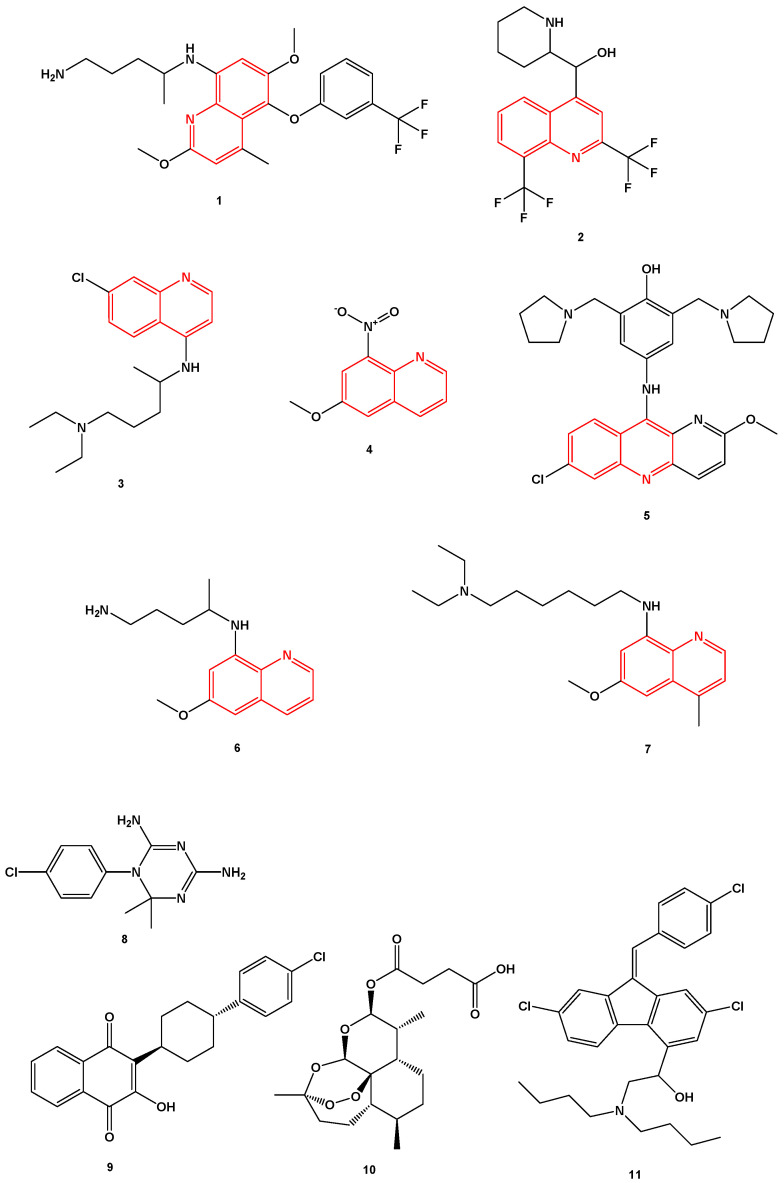
Chemical structures of the antimalarial drugs used in combination with antineoplastic in this work. Several compounds share a heterocyclic aromatic organic motif called quinoline (in red). Tafenoquine (**1**), mefloquine (**2**), chloroquine (**3**), 6-methoxy-nitroquinoline (**4**), pyronaridine (**5**), primaquine (**6**), sitamaquine (**7**), cycloguanil (**8**), atovaquone (**9**), artesunate (**10**), and lumefantrine (**11**).

In this work, we developed a new therapeutic strategy for breast cancer, based on the combination of an antineoplastic agent and several repurposed drugs. Drug repurposing is a rapid methodology that has already been used in clinic and allows to identify new uses for drugs that are already commercialized in addition to its original medical indication [29]. This strategy is advantageous over the development of new drugs, as it presents less risk of failure, since the pharmacokinetic and toxicological profiles of the drug are already known, and reduces development time and consequently represents less investment for pharmaceutical companies [30]. This strategy is also useful in the discovery of new therapeutic targets and metabolic pathways [31]. Antimalarial drugs have been shown to be potentially useful in the treatment of cancer [32,33,34,35]. The consideration of such drugs as possible anticancer agents relies on their ability to interfere with important oncogenic pathways, such as Wnt/β-catenin, STAT3, and NF-kB along with the emerging role of mitochondria in mediating the anti-tumor effects of antimalarials [36]. Among them, chloroquine, primaquine, and mefloquine particularly have been investigated in the treatment of numerous types of cancers, both alone and in combination with chemotherapy [37,38,39]. Our group selected several antimalarials and structurally grouped them into two main groups: Quinolinic-related derivatives (**1**–**7**, Scheme 1) and non-quinolinic-related derivatives (**8**–**11**, Scheme 1) and combined them with two antineoplastic drugs commonly used in breast cancer chemotherapy: DOX and PTX.

Mefloquine (**1**, Scheme 1) is a quinolinic analog commonly used in combination with artemisine in the treatment of malaria. Its mechanism of action is the inhibition of parasite protein synthesis by binding to the Pf80S subunit of the cytoplasmic ribosome of *Plasmodium falciparum* [40]. In cancer, mefloquine is believed to be involved in the modulation of the pathway induced by AMPK via ROS production, as well as in lysosomal disruption [41]. Mefloquine also presents antitumor effect and has already been tested on tumor cell lines of the breast [42], prostate [41], etc. In hormone receptor positive T47D and negative MDA-MB-231 breast cancer cell lines, mefloquine inhibited autophagy, triggered endoplasmic reticulum stress, and caused cell death [42]. 

Tafenoquine (**2**, Scheme 1) is an 8-aminoquinoline derivative that was recently approved (2018) for the treatment of malaria caused by the parasite *Plasmodium vivax*. This drug is a primaquine analogue and, although its mechanism remains unknown, it is thought that its pharmacological activity is due to the metabolization catalyzed by the CYP450 complex, namely by CYP2D6 [43].

Chloroquine (**3**, Scheme 1) is a 4-aminoquinoline approved for the treatment of malarial infections and later for the treatment of discoid and systemic lupus erythematosus and rheumatoid arthritis. Chloroquine has been extensively studied both in vitro and in vivo in various cancer types, as monotherapy and combination therapy [44]. The mechanisms of action of chloroquine against the malarial *Plasmodium* parasite are well known [45]. Several hypotheses have been proposed on how chloroquine exerts its anti-cancer effect, but some preclinical studies state that it influences the TLR9/nuclear factor kappa B (NF-κB) signaling pathway, the CXCL12/CXCR4 signaling pathway and the p53 pathway. Additionally, chloroquine might affect the autophagic flux at a late stage as it inhibits the fusion of the autophagosomes with the lysosomes and subsequent degradation of the autolysosome [44]. Several pre-clinical studies also support the use of chloroquine in anti-cancer therapy, mainly combined with conventional chemotherapeutic drugs, such as 5-fluorouracil [46,47]. Chloroquine has also been studied in different cancer cell types, such as lung [48], breast [49,50], and colon [51], resulting in anti-cancer effects. Indeed, more than 30 clinical studies are currently evaluating the activity of this antimalarial drug combined with various standard treatments in different types of cancer. The idea behind this strategy is that chloroquine can increase tumor cells sensibilization and therefore potentiate the therapeutic activity of chemotherapeutic drugs [44]. 

6-Methoxy-8-nitroquinoline (**4**, Scheme 1) was included in this study because it is also a quinoline derivative structurally very similar with the previous ones. No anticancer properties are reported for this drug in the literature. 

Pyronaridine (**5**, Scheme 1) is a quinoline-related drug that inhibits the formation of β-hematin and promotes the lysis of blood cells. Its mechanism of action as an anti-malaria agent is based on the inhibition of the DNA polymerase II of the parasite *P. falciparum* through the formation of a stable complex with the DNA. In breast tumor cells, this drug exhibits cytotoxicity through the induction of apoptosis, by interfering with the progression of the cell cycle [52]. 

Primaquine (**6**, Scheme 1) is a substituted quinoline used in the treatment of malaria, mainly administrated in combination with chloroquine. This drug is effective against all four malarial species that infect humans [53,54]. Its mechanism of action is not well understood but it is thought to interfere with the mitochondria of the parasite, by generating reactive oxygen species or by interfering with the electron transport [55]. In a study published by Kim et al., primaquine, in combination with mefloquine, has also demonstrate ability to sensitize drug-resistant KBV20C cancer cells by increasing P-glycoprotein inhibition [39].

Sitamaquine (**7**, Scheme 1), also known as WR6026, is an 8-aminoquinoline with excellent efficacy against parasites and currently undergoing phase 2b clinical trials for the treatment of visceral leishmaniasis [56,57]. Its mechanism of action involves inhibition of the respiratory chain complex II in parasites’ mitochondria, by triggering oxidative reactive species formation, ultimately leading to *Leishmania* parasites’ apoptosis [58]. 

Cycloguanil (**8**, Scheme 1) is the active metabolite of proguanil, a prophylactic antimalarial drug. Proguanil is often administered together with chloroquine and atovaquone in the treatment of resistant malaria. Cycloguanil inhibits the enzyme dihydrofolate reductase in *Plasmodium falciparum* and *Plasmodium vivax*, which is involved in the reproduction of the parasites [59]. 

Atovaquone (**9**, Scheme 1) is an antimalarial drug approved for clinical used by FDA in 2000. It is used for the prevention of malaria and in the treatment of pneumocystis pneumonia and/or toxoplasmosis in immune-compromised patients [60,61]. Atovaquone can be administered alone or in combination with Proguanil. Atovaquone is a quinone that targets the Co-enzyme Q10-dependence of mitochondrial complex III from *Plasmodium falciparum*, acting as a competitive inhibitor of co-enzyme Q10 [62]. Several studies demonstrate that atovaquone also has anti-tumoral activity against different types of cancer cells [63,64], namely breast cancer cells [65]. MCF-7 breast cancer cells treated with atovaquone demonstrate this drug inhibits oxygen-consumption and metabolically induces aerobic glycolysis and oxidative stress. Atovaquone can also be potent and selective in mixed populations of cancer stem cells (CSCs) and non-CSCs [65].

Artesunate (**10**, Scheme 1) is a derivative of artemisinin, which is also indicated in the treatment of malaria. This drug has a cytotoxic effect in different types of cells [66] and, specifically in gastric cancer, its effect is associated with a decrease in the expression of COX-2 [67]. In triple negative breast cells, studies have shown a decrease in cell proliferation through the blocking of cell cycle in G2/M (ROS-dependent) and in G1 (ROS-independent) [68]. In addition, the same study suggests this drug is capable of inhibiting angiogenesis, reducing invasion and metastasis as well as altering lysosomal mechanisms [68].

Lumefantrine (**11**, Scheme 1) is a synthetic amino alcohol fluorene derivative, related to mefloquine [69]. It is an antimalarial agent used in the treatment of acute uncomplicated malaria. Usually, it is administered in combination with artemether for improved efficacy. This combination acts against the erythrocytic stages of the malaria parasite [70]. It can also be used to treat infections caused by *Plasmodium falciparum* and unidentified *Plasmodium* species, including those acquired in chloroquine-resistant. The exact mechanism of action of lumefantrine remains unknown but available data suggest that this drug inhibits the formation of β-hematin and inhibits nucleic acid and protein synthesis [70].

In this work, we hypothesized that antimalarials could synergistically act with DOX and PTX in breast cancer treatment. Here, we propose a new combination model that consists of the combination of an antineoplastic and a repurposed drug. The main goal is to have a safe starting point, such as an antineoplastic drug, whose antitumor activity is guaranteed in tumor cells. The combination with the repurposed drug, which has an acceptable toxicological profile, aims to improve the activity of the reference drug, and simultaneously reduce its therapeutic dose. Since the toxicological profile of these drugs is already known, once their anti-cancer activity is better understood, the use of these drugs can be implemented clinically. Here, we demonstrate that the combination of DOX/PTX and some antimalarials such as artesunate, chloroquine, and mefloquine induces a greater anti-tumor effect compared to each drug alone in MCF-7 breast cancer cell line. These results should be confirmed in vivo and may be significant clinically. These data may lead to new therapeutic strategies for breast cancer therapy.

## 2. Materials and Methods 

### 2.1. Materials

Dulbecco’s modified Eagle’s medium (DMEM), fetal bovine serum (FBS), and penicillin-streptomycin solution were purchased from Millipore Sigma (Merck KGaA, Darmstadt, Germany). Other cell culture reagents were purchased from Gibco (Thermo Fisher Scientific, Inc, Waltham, MA, USA). Doxorubicin (cat. no. 15007), artesunate (cat. no. 11817), and cycloguanil (cat. no. 16861) were obtained from Cayman Chemical (Ann Arbor, Michigan, USA). Paclitaxel (taxol^®^; cat. no. 1097) and atovaquone (cat. no. 6358) was obtained from Tocris Bioscience (Bristol, UK). Pyronaridine (cat. no. P0049), tafenoquine (cat. no. SML0396), chloroquine (cat. no. C6628), lumefantrine (cat. no. L5420), primaquine (cat. no. 160393), sitamaquine (cat. no. SML1542), 6-methoxy-nitroquinoline (cat. no. 206571), Thiazolyl Blue Tetrazolium Bromide (MTT, cat. no. M5655) and sulforhodamine B (SRB, cat. no. S1402) were obtained from Sigma-Aldrich (Merck KGaA, Darmstadt, Germany). Mefloquine (cat. no. sc-211784) was purchased from Santa Cruz Biotechnology (Dallas, Texas, USA).

### 2.2. Cell Line and Cell Culture

Human breast cancer MCF-7 cell line was obtained from the American Type Culture Collection (ATCC; Virginia, USA) and maintained according to ATCC’s recommendations at 37 °C and 5% CO_2_ in DMEM medium supplemented with 10% fetal bovine serum, 100 U/mL penicillin G, and 100 µg/mL streptomycin. Cells were maintained in the logarithmic growth phase at all the times. The media was renewed every 2 days, trypsinized with 0.25% trypsin-EDTA, and subcultured in the same media. MCF-7 cells (10,000 cells/well) were seeded in 48-well plates and allowed to adhere overnight prior drug exposure. After that, the cell culture media were replaced with drug-containing media. Cells were exposed to drugs for 48 h, followed by SRB and MTT assays to evaluate single and combination drug treatments in the cell viability of these cells. 

### 2.3. Drug Treatment

The half maximal inhibitory concentration (IC_50_) value was first determined for each drug alone in MCF-cells. DOX concentrations ranged from 0.01 to 10 µM for the single-drug treatment. PTX concentrations ranged from 0.1 to 500 nM. Other drug concentrations ranged from 1 to 100 µM for the single-drug treatment. Combination studies were performed by combining DOX or PTX (Drug 1) with the repurposed drugs (Drug 2). Only drugs that present the most promising pharmacological profile such as artesunate, pyronaridine, tafenoquine, mefloquine, chloroquine, and cycloguanil were tested in combination with PTX and DOX. Both Drug 1 and Drug 2 concentrations were variable and the combination effects of equipotent concentrations (fixed ratio) of the IC_50_ values for each drug were evaluated.

### 2.4. Cell Viability Assay

To determine the effects of DOX, PTX, artesunate, pyronaridine, tafenoquine, mefloquine, atovaquone, chloroquine, cycloguanil, lumefantrine, primaquine, sitamaquine, and 6-methoxy-8-nitroquinoline on the viability of MCF-7 cells, MTT and SRB assays were used. For the MTT protocol, after drug treatment, the cell medium was removed, and 200 µL/well of MTT solution (0.5 mg/mL in PBS) was added. Cells were incubated for 3 h, protected from light. After this period, the MTT solution was removed, and DMSO (200 µL/well) was added to solubilize the formazan crystals. Absorbance was measured at 570 nm in an automated microplate reader (Sinergy HT, Biotek Instruments Inc., Winooski, VT, USA). For SRB assay, after treatments, the cultured cells were fixed with ice-cold 10% trichloroacetic acid for 30 min and stained with 0.4% SRB for 1h at room temperature. Excess dye was removed by rinsing several times with tap water. Protein-bound dye was dissolved with 400 µL 10 mM Tris base solution for the determination of absorbance with a microplate reader with a filter wavelength of 540 nm (Sinergy HT, Biotek Instruments Inc., Winooski, VT, USA). The IC_50_ of therapeutic drug was determined as each drug concentration showing 50% cell growth inhibition as compared with control. All conditions were performed in three times independently, in triplicate. 

### 2.5. Data Analysis

GraphPad Prism 8 (GraphPad Software Inc., San Diego, CA, USA) was used to produce concentration–response curves by nonlinear regression analysis. The viability of cells treated with each drug was normalized to the viability of control cells and cell viability fractions were plotted vs. drug concentrations in the logarithmic scale. 

### 2.6. Analysis of Drug Interactions

To quantify drug interaction between DOX/PTX and antimalarial drugs, the Combination Index (CI) was estimated by the unified theory, introduced by Chou and Talalay [71] using CompuSyn software (ComboSyn, Inc., New York, NY, USA). We used the mutually exclusive model, based on the assumption that drugs act through entirely different mechanisms [72]. The two drugs were combined in a fixed ratio of doses that correspond to 0.25, 0.5, 1, 2, and 4 times that of the individual IC_50_s. CI was plotted on y-axis as a function of effect level (Fa) on the x-axis to assess drug synergism between drug combinations. The CI is a quantitative representation of pharmacological interactions. CI < 1 indicates synergism, CI = 1 indicates additive interaction, and CI > 1 indicates antagonism. Experiments were conducted in triplicate (*n* = 3) with 3 replications at each drug concentration. 

### 2.7. Statistical Analysis

The results are presented as mean ± SEM for n experiments performed. All data were assayed in three independent experiences, in triplicate. Statistical comparisons between control and treatment groups, at the same time point, were performed with Student’s t test and one-way ANOVA test. Statistical significance was accepted at *p* values < 0.05. 

## 3. Results

### 3.1. Effect of DOX and PTX as Single Agents on MCF-7 Cellular Viability

**Figure 1:**
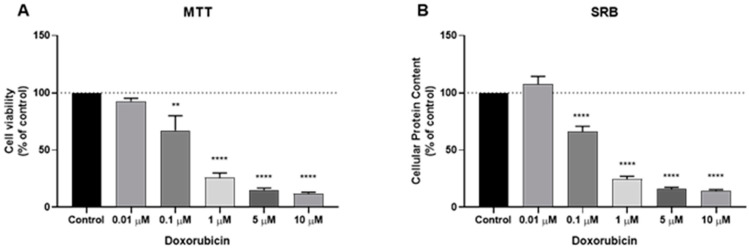
Effects of doxorubicin (DOX) on (**A**) cell viability and (**B**) cellular protein content. Cells were cultured in the presence of increasing concentrations of DOX. After 48 h MTT and SRB assays were performed to measured cellular viability as well as protein content. Values are expressed in percentage and represent means ± SEM. Each experiment was done three times independently (*n* = 3); ** statistically significant vs. control at *p* < 0.01. **** statistically significant vs. control at *p* < 0.0001.

We analyzed the cytotoxic potential of two established antineoplastic agents, namely PTX and DOX in a human breast cancer cell line MCF7 (noninvasive human breast cancer cell line, PR and ER-positive). To evaluate the effects of DOX and PTX alone on MCF-7 cells, the cells were treated with DOX alone (range 1–10 μM) and PTX alone (range 0.1–500 nM) for 48 h in MCF-7 cells. The percentage of cell survival was assessed by MTT assay and the percentage of cellular protein content was measured by SRB assay. The MTT assay is based on the measurement of mitochondrial activity, while the SRB is based on protein synthesis. The results of MTT and SRB tests agree with DOX activity and are given in Figure 1A,B, respectively. Our results reveal a significant activity of this drug at concentrations above 0.1 µM. The cells displayed strong response to the cytotoxic effect of DOX, with 1 µM killing almost 50% of cells.

**Figure 2:**
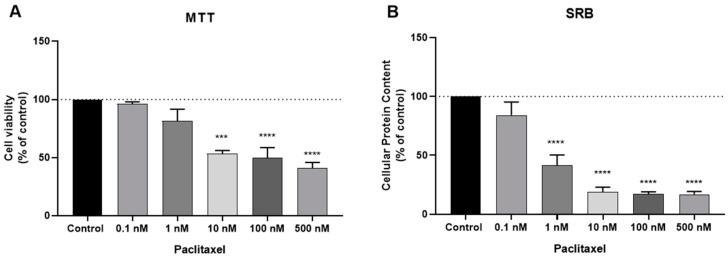
Effects of paclitaxel (PTX) on (**A**) cell viability and (**B**) cellular protein content. Cells were cultured in the presence of increasing concentrations of PTX. After 48 h MTT and SRB assays were performed to measured cellular viability as well as protein content. Values are expressed in percentage and represent means ± SEM. Each experiment was done three times independently (*n* = 3) *** statistically significant vs. control at *p* < 0.001. **** statistically significant vs. control at *p* < 0.0001.

PTX is an antineoplastic drug also used in breast cancer but has a totally different chemical structure from DOX and a different mechanism of action. While DOX is an anthracycline based on intercalation with DNA, PTX acts at the level of microtubules, with direct consequences on the cell cycle. The results concerning the PTX activity are given in Figure 2A,B for MTT and SBR assays, respectively, and demonstrate a great response of this drug for concentrations above 1 nM, with 10 nM causing the reduction of almost 50% of the cells. This response is more pronounced in the SRB assay, with a significant response from 1 nM. Together, these results support the anti-cancer activity of these two antineoplastic drugs in the treatment of breast cancer, namely in estrogen-receptor positive breast cancer and justify their use in the combinations proposed in this study.

### 3.2. Effect of Antimalarial Quinolinic Derivates as Single Agents on MCF-7 Cellular Viability

**Figure 3:**
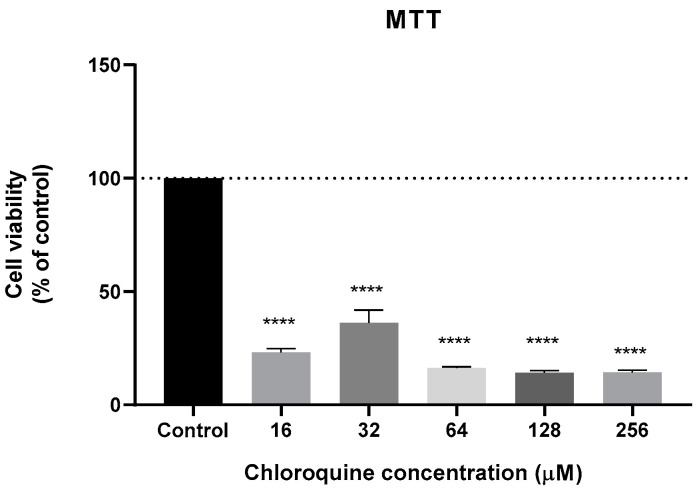
Effect of chloroquine in the cell viability of MCF-7 cells. Cells were cultured in the presence of increasing concentrations of chloroquine. After 48 h, MTT assay was performed to measured cellular viability. Values are expressed in percentage and represent means ± SEM. Each experiment was done three times independently (*n* = 3); **** statistically significant vs. control at *p* < 0.0001.

We next analyzed the cytotoxic potential of several antimalarial drugs, namely mefloquine, tafenoquine, 6-methoxy-nitroquinoline, pyronaridine, chloroquine, primaquine, and sitamaquine in a human breast cancer cell line MCF-7. As chloroquine anti-cancer activity has been evaluated several times in breast cancer cells, and its IC_50_ is well defined in the literature, the chloroquine cytotoxic effect was only evaluated by MTT assay and for a range of concentrations between 16 and 256 µM, i.e., tested for concentrations around its IC_50_ value described in the literature (64 µM) [50] (Figure 3). 

**Figure 4:**
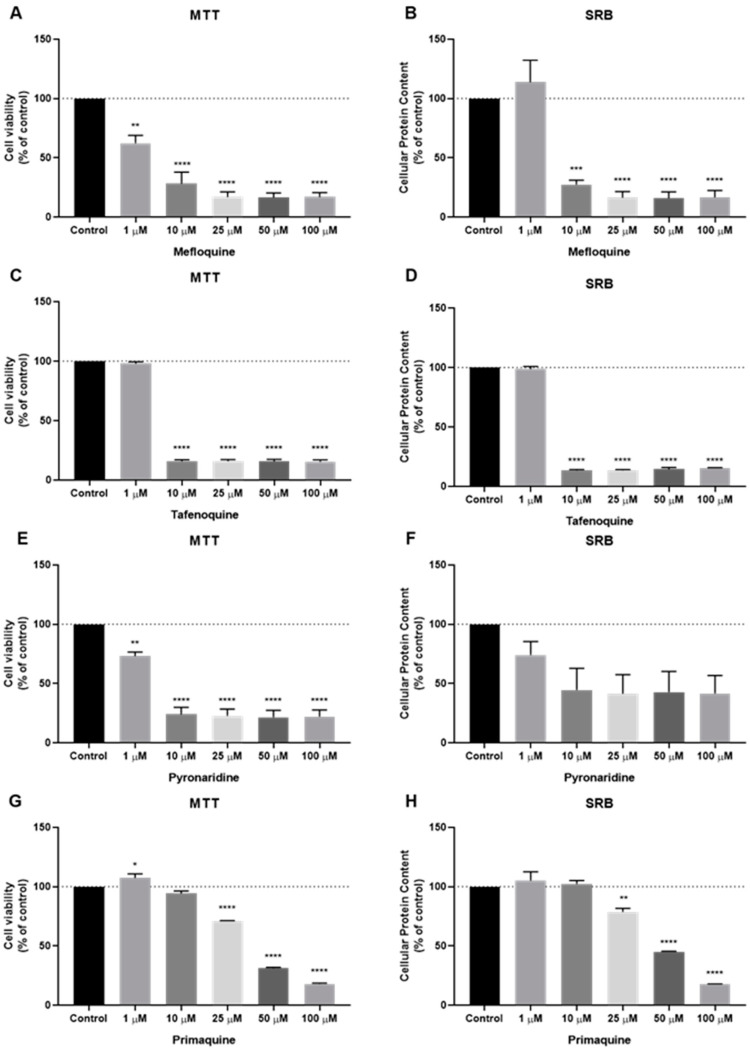
Effects of some antimalarial quinolinic derivates on MCF-7 cells. (**A**) Effect of mefloquine in cell viability and (**B**) protein content. (**C**) Effect of tafenoquine in cell viability and (**D**) protein content. (**E**) Effect of pyronaridine in cell viability and (**F**) protein content. (**G**) Effect of primaquine in cell viability and (**H**) protein content. Cells were cultured in the presence of increasing concentrations of each drug. After 48 h MTT and SRB assays were performed to measured cellular viability as well as protein content. Values are expressed in percentage and represent means ± SEM. Each experiment was done three times independently (*n* = 3); * statistically significant vs. control at *p* < 0.05. ** statistically significant vs. control at *p* < 0.01. *** statistically significant vs. control at *p* < 0.001. **** statistically significant vs. control at *p* < 0.0001.

The cells were then treated with increasing concentrations of each antimalarial drug (mefloquine, tafenoquine, 6-methoxy-nitroquinoline, pyronaridine, primaquine. and sitamaquine), starting from 1 µM to 100 µM to evaluate cell viability and cellular protein content after 48 h treatment, by MTT and SRB assays, respectively. These antimalarial drugs were grouped and chosen because they share a common structural feature: A quinolinic ring (**1**–**7**, Scheme 1). Based on the MTT assay, cytotoxic effects of mefloquine (Figure 4A) were significant even in concentrations of 1 µM, with higher concentrations causing a reduction of more than 50% of the cells (Figure 4A). SRB results show a significant decrease in protein content inside these cells (Figure 4B). Both MTT and SRB assays for tafenoquine treatment demonstrate a strong cytotoxic effect of this antimalarial drug in MCF-7 cells for all concentrations tested above 10 µM (Figure 4C,D). Pyronaridine treatment also significantly decreased cell viability, from 1 µM to 100 µM, with a pronounced effect for all concentrations above 10 µM (Figure 4E). SRB results are not significative but demonstrate the same tendency as MTT results (Figure 4F). MCF-7 cells treated with primaquine at doses up to 10 µM for 48 h, had little effect on cell viability and cellular protein content (Figure 4G,H). Above 50 µM, this drug caused a reduction of more than 50% of cell viability and protein synthesis. All concentrations showed a strong effect in the cell viability of MCF-7 cells, with more than 50% of the cells not viable. MTT and SRB assays for 6-methoxy-nitroquinoline and sitamaquine demonstrate a lack of efficacy of these antimalarial drugs on the reduction of cell viability and protein content (Figure S1).

### 3.3. Effect of Antimalarial Non-Quinolinic Derivates as Single Agents on MCF-7 Cellular Viability

**Figure 5:**
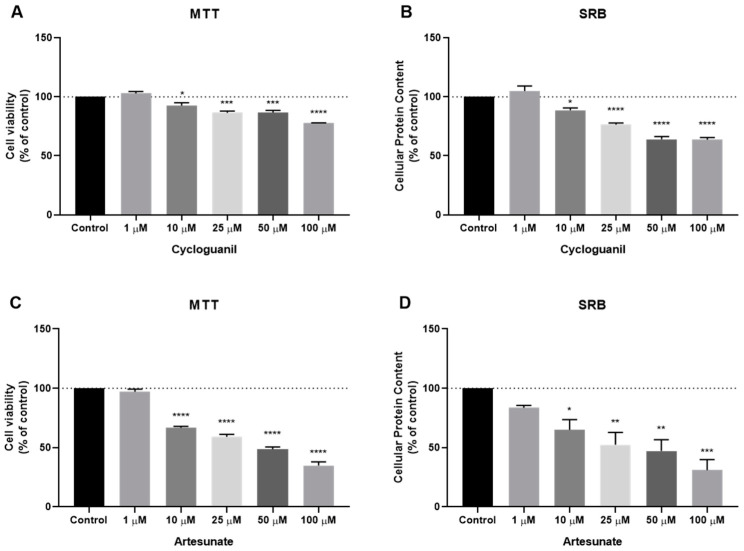
Effects of some antimalarial non-quinolinic derivates on MCF-7 cells. (**A**) Effect of cycloguanil in cell viability and (**B**) protein content. (**C**) Effect of artesunate in cell viability and (**D**) protein content. Cells were cultured in the presence of increasing concentrations of each drug. After 48 h MTT and SRB assays were performed to measured cellular viability as well as protein content. Values are expressed in percentage and represent means ± SEM. Each experiment was done three times independently (*n* = 3); * statistically significant vs. control at *p* < 0.05. ** statistically significant vs. control at *p* < 0.01. *** statistically significant vs. control at *p* < 0.001. **** statistically significant vs. control at *p* < 0.0001.

We also evaluated the cytotoxic potential of several antimalarial non-quinolinic derivates, namely artesunate, lumefantrine, cycloguanil and atovaquone. The cells were treated as previous described. Based on MTT and SRB results, cytotoxic (Figure 5A) and protein synthesis effects (Figure 5B) of cycloguanil were significant for concentrations above 10 µM, but the effect was less pronounced than the previous family of antimalarial drugs. Artesunate treatment demonstrated a higher reduction in cell viability (Figure 5C), in concentrations above 10 µM, to a higher extent than cycloguanil, with a reduction of more than 50% of cell viability in concentrations of 50 µM or more. SRB results for artesunate treatment are similar to those obtained by the MTT assay (Figure 5D). Neither atovaquone nor lumefantrine demonstrate good efficacy against MCF-7 cells, both in MTT and SRB assays (Figure S2).

Based on the results obtained in the MTT assay, we estimated the IC_50_ for both DOX and PTX in MCF-7 cells. The dose-response graph estimates the IC_50_ for DOX and PTX to be about 0.2 µM and 3 nM, respectively (Figure S3). Given the high antitumor activity of these drugs at very low doses, these antineoplastics were considered good candidates for the combination with antimalarial drugs.

After finding the most promising antimalarial drugs, based on the previous MTT and SRB results, we evaluated the IC_50_ for each drug to further use in combination with DOX and PTX. At this stage, the most promising antimalarials were mefloquine, tafenoquine, primaquine, pyronaridine, chloroquine, artesunate, and cycloguanil. The first five are antimalarial quinolinic derivates and the last two are non-quinolinic derivatives. MTT-based dose-response curves are shown in Figure S4. Treatment with pyronaridine for 48 h resulted in the higher reduction of viable cells, with an IC_50_ of about 1.5 µM. Mefloquine, pyronaridine, and tafenoquine presented an IC_50_ lesser than 10 µM, with an IC_50_ around 1 µM, 1.5 µM, and 3 µM, respectively. Artesunate showed less cytotoxic activity than tafenoquine, with an IC_50_ around 12 µM. Both cycloguanil and primaquine presented less ability than other antimalarial drugs to decrease cell viability of MCF-7 cells, which resulted in higher IC_50_. Cycloguanil and primaquine IC_50_ were around 20 µM and 30 µM, respectively. In general, the selected antimalarials showed great activity in reducing the cell viability of breast cancer cells, with very low IC_50_. These results demonstrate antimalarial drugs are good candidates for use in combination with PTX and DOX. Table 1 summarizes the IC_50_ of all drugs used in the further combinations. 

### 3.4. Effect of Various Combinations of DOX and Different Antimalarials in MCF-7 Cells

**Figure 6:**
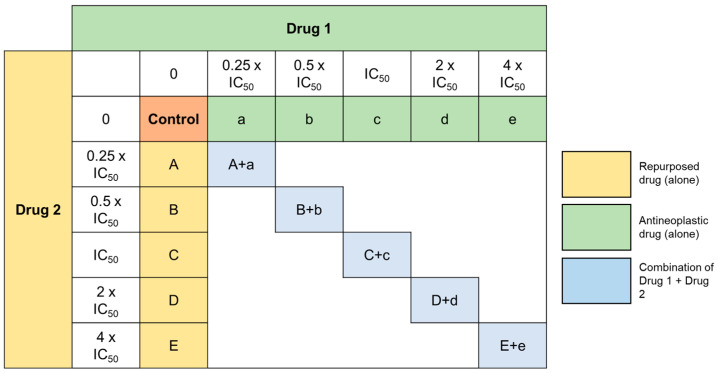
Representation of the design of the combination experiences. Drug 1 corresponds to the antineoplastic agent and Drug 2 corresponds to antimalarial drugs. Cells were treated with Drug 1 and Drug 2 alone and in combination in a fixed ration for 48 h, in the concentrations of 0.25 × IC_50_, 0.5 × IC_50_, IC_50_, 2 × IC_50_, and 4 × IC_50_.

After finding the best candidates for repurposing in breast cancer therapy and their corresponding IC_50_, we designed a model of combination consisting by one antineoplastic drug, DOX and each antimalarial drug. MCF-7 cells were treated with the two drugs alone and combined in a fixed ratio, in the concentrations of 0.25 × IC_50_, 0.5 × IC_50_, IC_50_, 2 × IC_50_, and 4 × IC_50_ as represented in Figure 6. We evaluated the combinations of DOX + Artesunate, DOX + Mefloquine, DOX + Tafenoquine, DOX + Pyronaridine, and DOX + cycloguanil by MTT assay. As chloroquine has been widely tested in several cell lines, we also included the combination of DOX + chloroquine in this study, with the IC_50_ based on the literature [50]. 

**Figure 7:**
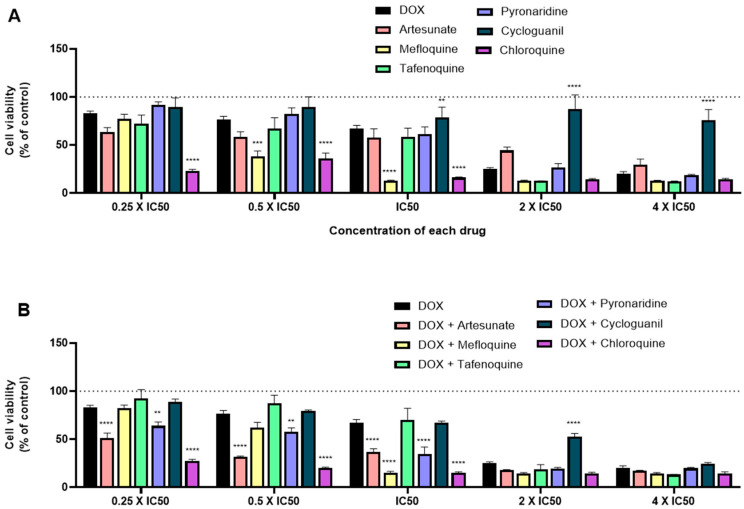
Growth inhibition of MCF-7 cells after 48 h of single drug (**A**) and combination therapy (**B**) exposure. Cells were exposed to concentrations of each drug of 0.25, 0.5, 1, 2, and 4 times their IC_50_ and cell viability was evaluated by MTT assay. The drugs in combination were co-administered at the same time. Values are expressed in percentage in relation to DOX activity and represent means ± SEM. Each experiment was done three times independently (*n* = 3); ** statistically significant vs. control at *p* < 0.01. *** statistically significant vs. control at *p* < 0.001. **** statistically significant vs. control at *p* < 0.0001.

When tested alone, and in line with the previous results, mefloquine and chloroquine treatment resulted in higher cytotoxic activity, with significant decrease of cell viability, mainly for the concentrations of IC_50_ and half of their IC_50_, with better results than DOX alone. When tested for 2 × IC_50_, almost all drugs displayed great anti-cancer activity, with the exception of cycloguanil (Figure 7A). When combined with DOX and comparing to DOX alone, almost all combinations resulted in enhanced anti-cancer effects, with DOX + mefloquine and DOX + chloroquine being the most promising combinations for the concentration of IC_50_, which is in line with the results for each drug alone. Combinations of DOX + artesunate and DOX + pyronaridine also resulted in less viable MCF-7 cells than DOX alone, but to a lesser extent than the previous combinations (Figure 7B). Combination of DOX + artesunate, DOX + pyronaridine, and DOX + chloroquine in concentrations below the IC_50_ of each drug resulted in significant decreases of cell viability compared to DOX alone. 

### 3.5. Effect of Various Combinations of PTX and Different Antimalarials in MCF-7 Cells

**Figure 8:**
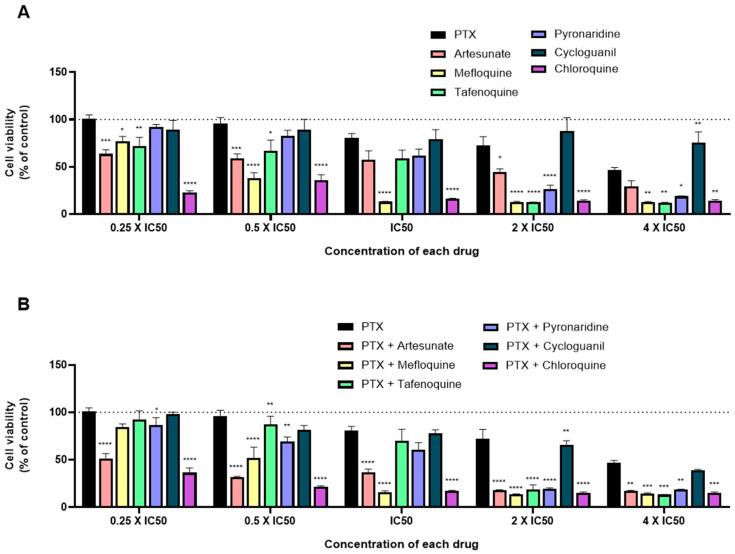
Growth inhibition of MCF-7 cells after 48 h of single drug (**A**) and combination therapy (**B**) exposure. Cells were exposed to concentrations of each drug of 0.25, 0.5, 1, 2, and 4 times their IC_50_ and cell viability was evaluated by MTT assay. The drugs in combination were co-administered at the same time. Values are expressed in percentage in relation to PTX activity and represent means ± SEM. Each experiment was done three times independently (*n* = 3); * statistically significant vs. control at *p* < 0.05. ** statistically significant vs. control at *p* < 0.01. *** statistically significant vs. control at *p* < 0.001. **** statistically significant vs. control at *p* < 0.0001.

To evaluate the influence of the antineoplastic agent, we evaluated the same antimalarial drugs with PTX. Drugs were also tested in MCF-7 cells both alone and combined in the concentrations of 0.25 × IC_50_, 0.5 × IC_50_, IC_50_, 2 × IC_50_, and 4 × IC_50_ for 48 h. Cell viability was measured by the MTT assay. Comparing to DOX, PTX demonstrated a higher efficacy in decreasing cell viability, which resulted in a lower IC_50_ of around 3 nM, comparing to 0.1 µM for DOX (Figure 8A). Although it has a lower IC_50_, when tested alone for all concentrations, this drug does not affect MCF-7 cell viability to a larger extent than DOX. When compared to PTX activity, mainly for higher doses (2 × IC_50_ and 4 × IC_50_), almost all antimalarial drugs demonstrate significant anti-cancer activity, with mefloquine, tafenoquine, and chloroquine having the best profiles for further combination (Figure 8B). Indeed, when combined with PTX in higher doses (2 × IC_50_ and 4 × IC_50_), all antimalarials resulted in a significant reduction of viable cells, with the exception of cycloguanil. For lower doses, the combinations of PTX with artesunate, mefloquine, and cycloguanil are the most promising ones. Comparing antimalarial drugs combinations with DOX and PTX, the last ones seem to be most effective for breast cancer therapy. 

### 3.6. Synergistic Combinations of Antineoplastic and Antimalarial Drugs in MCF-7 Cells

**Figure 9:**
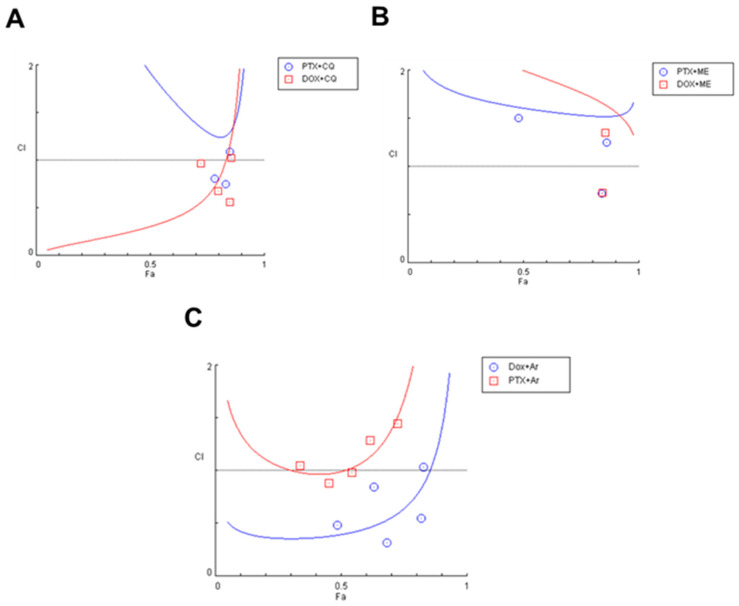
Chou-Talalay method Fa-CI plot of DOX/PTX and chloroquine (**A**), mefloquine (**B**) and artesunate (**C**). CI was plotted on y-axis as a function of effect level (Fa) on the x-axis to assess drug synergism. CI < 1, CI = 1 and CI > 1 indicate synergism, additivity, and antagonism, respectively.

**Figure 10:**
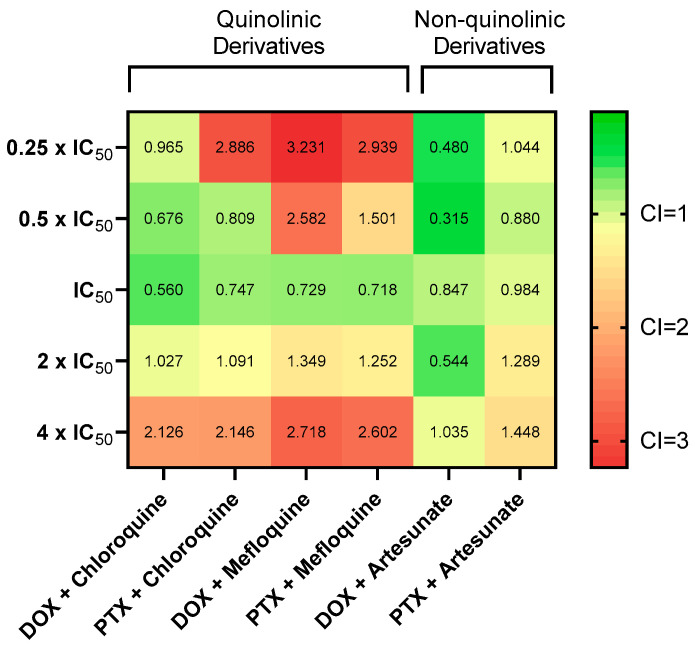
Synergy heat map compiling CI values. The squares in the figure indicate antagonism (CI > 1.2, red), additivity (0.9 < CI < 1.1, yellow), and synergism (CI < 0.9, green) between DOX/PTX and chloroquine/mefloquine/artesunate against MCF-7 cells at combination settings described in Figure 6.

To investigate the effects of combined the antineoplastic agents DOX and PTX with the previous antimalarial agents, and after finding the most promising ones based on MTT assays, the combination index (CI) was calculated using the Chou-Talalay method. CI was plotted on y-axis as a function of effect level (Fa) on the x-axis to assess drug synergism. The fractional effect is a value between 0 and 1, where 0 means the drug had no effect on cell viability and 1 means the drug produced full effect on decreasing cell viability. Results for synergistic combinations are summarized in Table 2. Combination of DOX + chloroquine demonstrated more synergism than the combination with PTX. For lower doses of 0.05 µM + 16 µM, 0.1 µM + 32 µM, and 0.2 µM + 64 µM, corresponding to the concentrations of DOX and chloroquine, respectively, all combinations were synergic and produced effects between 0.724 and 0.851. The combination of 32 and 64 µM with 1.5 nM and 3 nM, respectively, also resulted in synergism, with Fa between 0.782 and 0.831 (Figure 9A). The combination of the IC_50_s of DOX + mefloquine (8 µM and 0.2 µM, respectively) for 48 h resulted in a synergistic effect (CI < 1), with a fractional effect of 0.845. The combination of 3 nM of PTX and 8 µM of mefloquine also resulted in synergism, with a fractional effect of 0.841 (Figure 9B). Combination of artesunate with DOX resulted in more synergism than the combination of PTX with the same antimalarial drug, with CI < 1 for almost all pairs of concentrations, with effects between 0.487 and 0.821. Only the combinations of 6 and 12 µM of artesunate with 1.5 and 3 nM of PTX were synergic but produced lower fractional effect (Figure 9C). Together, these results demonstrate non-quinolinic antimalarial derivates may be more promising to evaluate in future combinations (Figure 10). Other combinations were also performed, but the results were not significative, and are described in the Supplementary Material (Figure S5). 

## 4. Discussion

Drug repurposing has been gaining attention among scientific community, as a faster and cheaper strategy to identify new potential anti-cancer therapies. These drugs had previously been approved for another disease and have pharmacokinetics, pharmacodynamics, and toxicological profiles well established, facilitating their introduction in clinical practice. Drug combination is a strategy that allows the reduction of drug concentration needed for therapeutical effects. While many studies have identified synergistic drug pairs for specific cancer cells based on the combination of antineoplastic agents, few reports if antimalarials offers improved therapeutic benefits in drug combination with antineoplastic agents. Even though current protocols of breast cancer treatment are quite effective, current strategies reside in the dose limiting toxicity of the antineoplastic drugs that are used as an adjuvant therapy [73]. DOX and PTX are essential agents in the treatment of breast cancer but their use results in severe side effects and drug resistance, thereby limiting maximum benefits achievement. Despite the good results of DOX and PTX in breast cancer chemotherapy, side effects such as cardiotoxicity and acquire resistance are still a major problem. To overcome tumor resistance, chemotherapy is usually based in higher doses and long-term use of antineoplastic drugs, so current studies aim to decrease the dose and time of exposure to these drugs. More and more, research is being made in the investigation of new drugs that can work synergistically with antineoplastic drugs, but less investigation has been made using repurposed drugs, namely antimalarials. Some studies have been evaluating the combination of chemotherapeutic agents with different drug classes to improve breast cancer patients lives [7,74,75].

Pre-clinical studies suggest that antimalarial drugs can have intrinsic anti-cancer activity, decrease cellular proliferation and induce apoptosis [33,35,39,49,50,76,77]. Experimental evidences suggest that some antimalarials exhibit synergism with other drugs with no increased toxicity toward normal cells. In this regard, we studied the potential synergistic effects of several antimalarials with DOX and PTX, chemotherapeutic agents for treatment of breast cancer. The antitumor activity of antimalarials as combination drugs with DOX and PTX is compared to that of two usually used chemotherapeutic agents, DOX and PTX alone in a breast cancer cell line. To identify the best candidates for repurposing, several antimalarials were screened to treat MCF-7 cells. After evaluation of cytotoxic activity, seven antimalarials were selected for combination with DOX and PTX. For each drug, IC_50_ was determined and cells were treated with the concentrations of 0.25, 0.5, 1, 2 and 4 times the IC_50_ of each drug, alone and in combination. Based on these results, synergism was evaluated using the Chou–Talalay method, which is based on the median-effect equation, derived from the mass-action law principle. This unified theory encompasses the Michaelis–Menten, Hill, Henderson–Hasselbalch, and Scatchard equations in biochemistry and biophysics and provides a quantitative definition for additive effect (CI = 1), synergism (CI < 1), and antagonism (CI > 1) in drug combinations [71]. Our results demonstrated that antimalarial drugs as single agents have ability to decrease cell viability in a concentration-dependent manner and in combination can improve the anti-cancer activity of DOX and PTX in ER-positive breast cancer cells. We demonstrated for the first time that the combination of artesunate, mefloquine, and chloroquine can synergistically inhibit breast cancer proliferation in MCF-7 cells. 

These results imply that antimalarial drugs may be a promising chemosensitizing compound for enhancing the cytotoxic effects of DOX and PTX in MCF-7 cells. Since these antimalarials are already clinically available, its use in clinical practice for anti-cancer therapy is feasible. Since different breast cancer cell lines are metabolically different, have specific phenotypic characteristics and genotypic status, and different drug resistance patterns, further research should be made in other breast cancer cells, such as MDA-MB-231 and SUM159 cells (triple negative). Based on previous studies [36], we believe antimalarials may interfere in important oncogenic pathways such as Wnt/β-catenin, STAT3, NF-kB, and in mitochondria metabolism, leading to the formation of ROS, which sensitizes cancer cells and enhances antineoplastic drugs’ activity; however, deeper mechanistic studies are recommended for further evaluation of the anticancer mechanisms underlying these combinations. These combinations should also be investigated for another types of cancer, such as pancreatic, colorectal, etc. 

## 5. Conclusions

There have been extensive efforts to enhance the efficacy of chemotherapy and find synergistic drug combinations for cancer therapy. This novel combination model consisting of antimalarials and antineoplastic drugs demonstrated that a combined treatment can be advantageous for cancer therapy that it is ineffective or too toxic when chemotherapeutics alone are used. These results are encouraging for further research that considers antimalarials as chemosensitizing agents of DOX or PTX. In summary, we demonstrated the repurposing potential of antimalarials and found synergy between DOX/PTX and artesunate, mefloquine, and chloroquine, which makes these combinations a promising approach to treat breast cancer, mainly the non-invasive and estrogen-receptor positive ones. The drugs used in combination may have independent mechanisms and may be a promising strategy that is better than suppressing only one cancer resistance pathway. These results suggest that these combinations might be a promising approach to treat breast cancer. More studies are needed to identify the anticancer mechanisms underlying these combinations and to identify the cell signaling pathways. These combinations should also be investigated in another breast cancer cells, such as triple negative or in another types of cancer, for a broader application. In addition, in vivo studies should be taken to explore further these effects and evaluate the safety and efficacy of the drug combinations studied in this model.

## Figures and Tables

**Table 1:** Cytotoxicity of DOX/PTX and some antimalarials in MCF-7 breast cancer cells. IC_50_ values are given as mean.

Drug	IC_50_/µM
DOX	0.1699 ± 0.0924
PTX	0.003 ± 0.0001
Mefloquine	1.241 ± 0.064
Tafenoquine	2.595 ± 0.060
Primaquine	29.90 ± 0.02
Pyronaridine	1.388 ± 0.216
Artesunate	11.60 ± 0.04
Cycloguanil	20.30 ± 0.08

**Table 2:** CI values and respective fractional effect of various combinations of DOX/PTX and mefloquine, chloroquine and artesunate. CI in red indicate concentrations of drug pairs that are synergic.

Combination (Drug A + Drug B)	Concentration (Drug A + Drug B)	Fractional Effect(Fa)	CI
DOX + chloroquine	0.05 µM + 16 µM 0.1 µM + 32 µM 0.2 µM + 64 µM 0.4 µM + 128 µM 0.8 µM + 256 µM	0.724 0.797 0.851 0.856 0.854	0.965 0.676 0.560 1.027 2.126
PTX + chloroquine	0.75 nM + 16 µM 1.5 nM + 32 µM 3 nM + 64 µM 6 nM + 128 µM 12 nM + 256 µM	0.636 0.782 0.831 0.850 0.851	2.886 0.809 0.747 1.091 2.146
DOX + mefloquine	0.05 µM + 2 µM 0.1 µM + 4 µM 0.2 µM + 8 µM 0.4 µM + 16 µM 0.8 µM + 32 µM	0.176 0.375 0.845 0.856 0.855	3.231 2.582 0.729 1.349 2.718
PTX + mefloquine	0.75 nM + 2 µM 1.5 nM + 4 µM 3 nM + 8 µM 6 nM + 16 µM 12 nM + 32 µM	0.158 0.479 0.841 0.863 0.857	2.939 1.501 0.718 1.252 2.602
DOX + artesunate	0.05 µM + 3 µM 0.1 µM + 6 µM 0.2 µM + 12 µM 0.4 µM + 24 µM 0.8 µM + 48 µM	0.487 0.684 0.631 0.821 0.828	0.480 0.315 0.847 0.544 1.035
PTX + artesunate	0.75 nM + 3 µM 1.5 nM + 6 µM 3 nM + 12 µM 6 nM + 24 µM 12 nM + 48 µM	0.337 0.453 0.544 0.617 0.726	1.044 0.880 0.984 1.289 1.448

## Supplementary Materials

The following are available online at https://www.mdpi.com/2218-273X/10/12/1623/s1, Figure S1: Effects of 6-methoxy-nitroquinoline (A and B) and sitamaquine (C and D) on MCF-7 cells. Figure S2: Effects of lumefantrine (A and B) and atovaquone (C and D) on MCF-7 cells. Figure S3: Concentration-response curves of DOX (A) and PTX (B) on MCF-7 cells based on results obtained by MTT assay. Figure S4: Concentration-response curves of mefloquine (A), tafenoquine (B), primaquine (C), pyronaridine (D), artesunate (E) and cycloguanil (F) on MCF-7 cells based on results obtained by MTT assay. Figure S5: Chou-Talalay method Fa-CI plot of DOX/PTX and tafenoquine (A) and pyronaridine (B).
