# Supplementary material for: New Trends for Antimalarial Drugs: Synergism between Antineoplastics and Antimalarials on Breast Cancer Cells
Source: Biomolecules. 2020 Dec 1;10(12):1623. doi: 10.3390/biom10121623 (PMC7761440; doi:10.3390/biom10121623)
Supplement: Supplementary file 1 [file biomolecules-10-01623-s001.pdf]

New trends for antimalarial drugs: synergism between antineoplastics and antimalarials on breast cancer cells

Diana Duarte [1,2] and Nuno Vale [3,*]

1 OncoPharma Research Group, Center for Health Technology and Services Research (CINTESIS), Rua Dr. Plácido da Costa, 4200-450 Porto, Portugal
2 Faculty of Pharmacy of University of Porto, Rua Jorge Viterbo Ferreira, 228, 4050-313 Porto, Portugal
3 Faculty of Medicine, University of Porto, Al. Prof. Hernâni Monteiro, 4200-319 Porto, Portugal

* Correspondence: nunovale@med.up.pt;

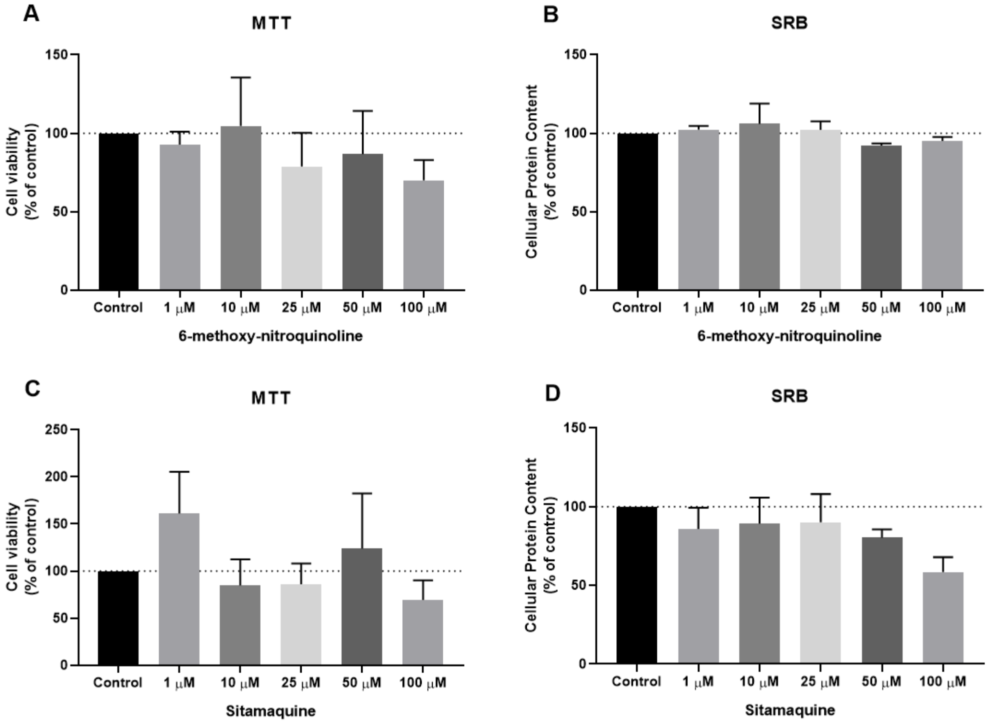

Figure S1. Effects of 6-methoxy-nitroquinoline (**A** and **B**) and sitamaquine (**C** and **D**) on MCF-7 cells. Cells were cultured in the presence of increasing concentrations of each drug. After 48 h MTT and SRB assays were performed to measured cellular viability as well as protein content. Values are expressed in percentage and represent means ± SEM. Each experiment was done three times independently (n=3).

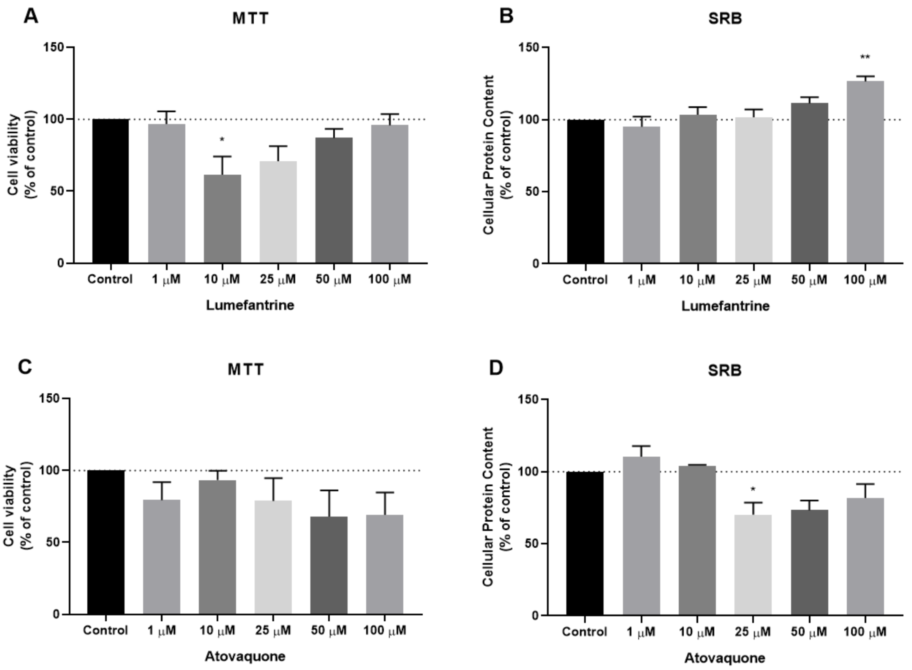

Figure S2. Effects of lumefantrine (**A** and **B**) and atovaquone (**C** and **D**) on MCF-7 cells. Cells were cultured in the presence of increasing concentrations of each drug. After 48 h MTT and SRB assays were performed to measured cellular viability as well as protein content. Values are expressed in percentage and represent means ± SEM. Each experiment was done three times independently (n=3); *statistically significant vs. control at $p < 0.05$. **statistically significant vs. control at $p < 0.01$.

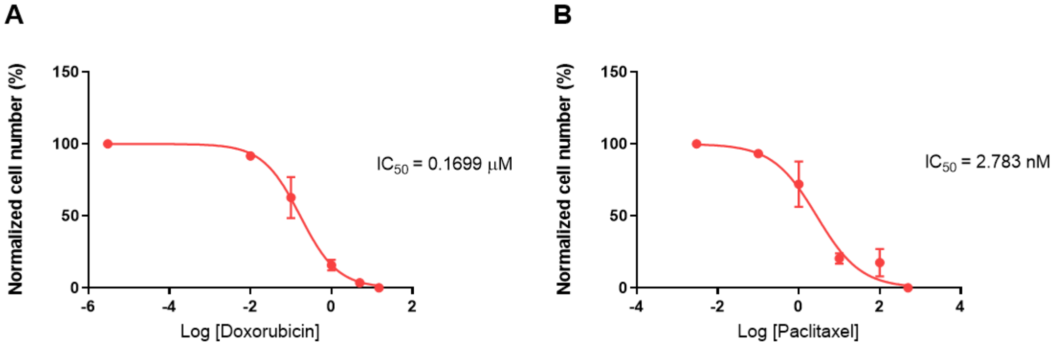

Figure S3. Concentration-response curves of DOX (**A**) and PTX (**B**) on MCF-7 cells based on results obtained by MTT assay. The results represent the mean ± SEM of three independent experiments performed in triplicate.

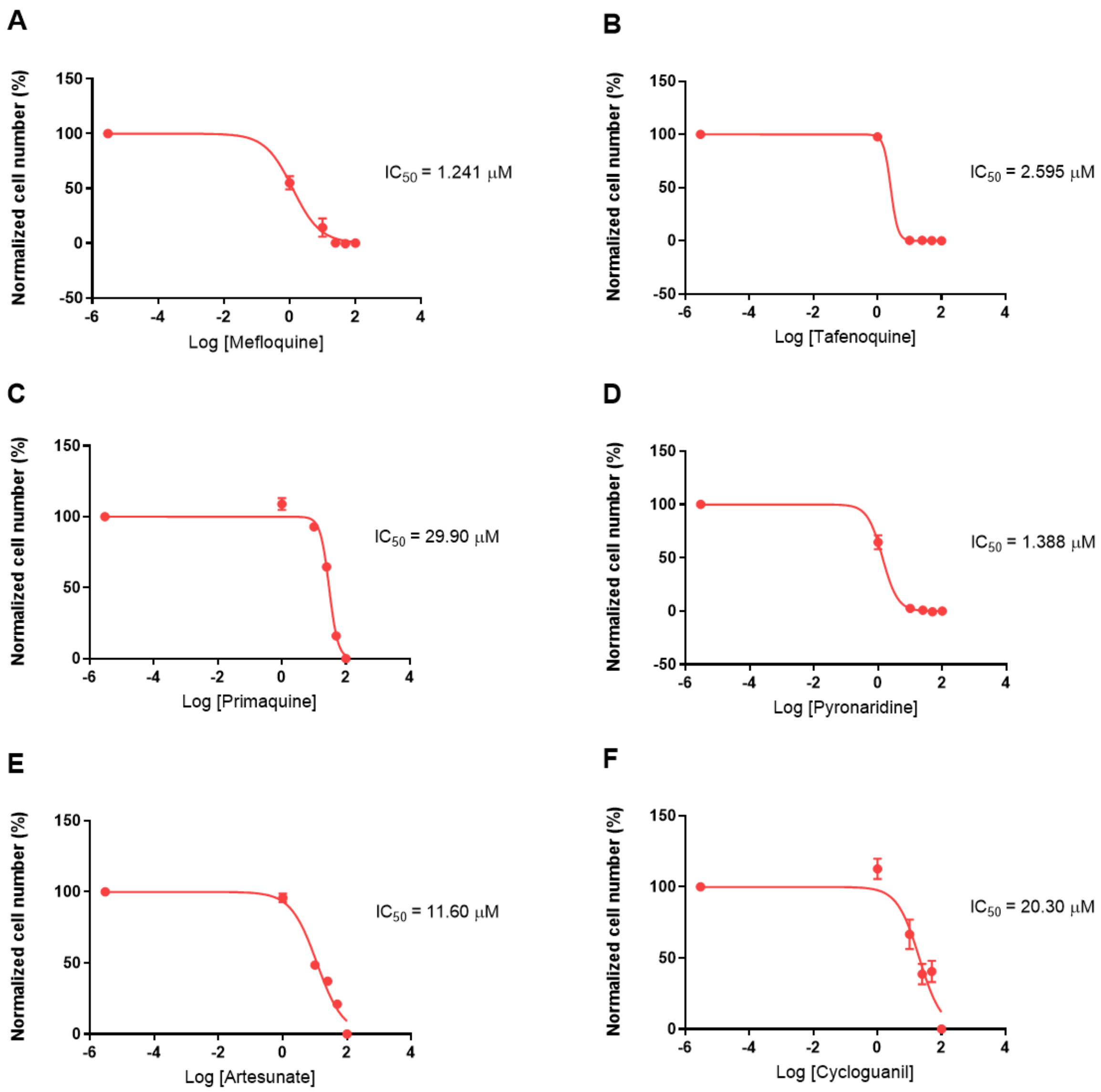

Figure S4. Concentration-response curves of mefloquine (**A**), tafenoquine (**B**), primaquine (**C**), pyronaridine (**D**), artesunate (**E**) and cycloguanil (**F**) on MCF-7 cells based on results obtained by MTT assay. The results represent the mean ± SEM of three independent experiments performed in triplicate.

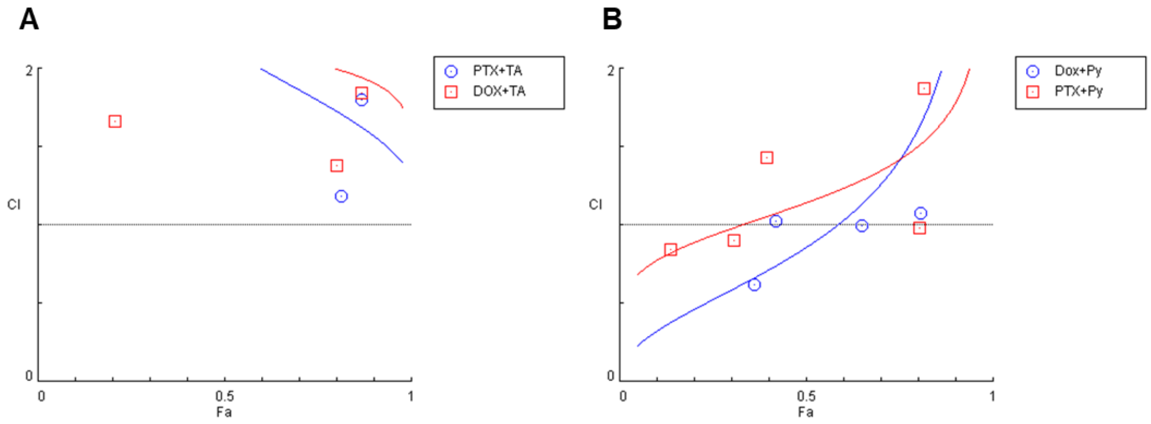

Figure S5. Chou-Talalay method Fa-CI plot of DOX/PTX and tafenoquine (**A**) and pyronaridine (**B**). CI was plotted on y-axis as a function of effect level (Fa) on the x-axis to assess drug synergism. CI<1, CI=1 and CI>1 indicate synergism, additivity, and antagonism, respectively.
